# Impact of Hydrothermal Treatments on Nutritional Value and Mineral Bioaccessibility of Brussels Sprouts (*Brassica oleracea* var. *gemmifera*)

**DOI:** 10.3390/molecules27061861

**Published:** 2022-03-13

**Authors:** Joanna Doniec, Adam Florkiewicz, Robert Duliński, Agnieszka Filipiak-Florkiewicz

**Affiliations:** 1 Department of Plant Products Technology and Nutrition Hygiene, Faculty of Food Technology, University of Agriculture in Krakow, 122 Balicka Street, 30-149 Krakow, Poland; agnieszka.filipiak-florkiewicz@urk.edu.pl; 2 Department of Food Analysis and Quality Assessment, Faculty of Food Technology, University of Agriculture in Krakow, 122 Balicka Street, 30-149 Krakow, Poland; adam.florkiewicz@urk.edu.pl; 3 Department of Biotechnology and General Technology of Food, Faculty of Food Technology, University of Agriculture in Krakow, 122 Balicka Street, 30-149 Krakow, Poland; robert.dulinski@urk.edu.pl

**Keywords:** Brussels sprouts, boiling, steaming, sous vide, composition, bioaccessibility

## Abstract

Hydrothermal treatment of Brussels sprouts (*Brassica oleracea* var. *gemmifera*) induces both physical and chemical changes in nutrients and non-nutrients. It also affects the bioaccessibility of individual compounds. The aim of this study was to investigate the influence of hydrothermal treatment (boiling, steaming, and sous vide technique) on the concentration of the selected nutrients and non-nutrients in Brussels sprouts and in vitro bioaccessibility of the mineral components. It has been shown that, in terms of the leaching of nutrients and non-nutrients into the aqueous medium, traditional cooking in water involves the greatest percentage loss (the highest decrease in dry matter (11.8%), ash (13.3%), protein (10.4%), crude fat (43.3%), dietary fiber (9.5%), digestible carbohydrates (12.2%), and most of mineral components (7.6–39.8%)). In contrast, steam cooking and sous vide cooking of Brussels sprouts allow a higher level of preservation of the individual compounds. By using reduced process temperatures and vacuum packaging, sous vide cooking can be an alternative to traditional cooking to preserve the higher nutritional value of *Brassica oleracea* var. *gemmifera* (preservation of dry matter, ash, crude fat, and most of the mineral components at the level of the raw sample *p* ≤ 0.05).

## 1. Introduction

Diets with high consumption of plant products is increasingly becoming an interest of many consumers [1]. *Brassica* vegetables, offering a high concentration of minerals, vitamins, and bioactive substances while providing a low fat content, are considered to be plant-based products with health-promoting effects on the human body. Their consumption at a minimum level of five portions per week is recommended to complement the balanced diet of adults [2]. *Brassica* vegetables are mainly consumed after heat treatment; hence, when analyzing their composition, it is important to consider the factors related to hydrothermal treatment, which have a direct effect on the concentration of specific nutrients and bioactive components contained in the vegetables [3]. Boiling in water, steaming, or the use of microwaves exert significant effects on the biochemical composition of *Brassica* vegetables. Researchers have confirmed that nutrients and non-nutritive compounds decrease in concentration in *Brassica* vegetables during conventional hydrothermal treatments [4,5,6,7,8,9,10]. The sous vide method, however, is an alternative preparation method for these products, allowing higher preservation of nutritional value of examined *Brassica* vegetables [4,5,6,7]. It involves the preparation of the products in sealed plastic vacuumized pouches and their heat treatment under strictly controlled temperature conditions. By carefully controlling the conditions of the heat treatment process and preparing the products in sealed and temperature-resistant pouches, it is possible to retain more nutritional content, extend the shelf life, and obtain an initial product with a better texture than traditional hydrothermal treatment methods. However, this method requires greater precision, longer processing times, prior vacuum packing, and precise temperature control [11].

Comprehensive determination of the concentration of essential nutrients and non-nutrients enables the nutritional characterization of the food [12]. However, research based only on the food products does not provide sufficient data on the concentrations of the individual components available for nutritional use by the human body after digestion. Such characteristics can be provided by studies based on an in vitro digestion model. Bioaccessibility studies carried out using in vitro digestion model have allowed researchers to define the scope of the influence of food processing on the biochemical composition of food products [13]. In vitro digestion is commonly used to study the bioaccessibility, changes in structure, and release of individual food components in laboratory-created simulated conditions of the human digestive system [14].

The aim of this study was to investigate the impact of hydrothermal treatment on the concentration of the selected nutrient and non-nutrient compounds of Brussels sprouts (*Brassica oleracea* var. *gemmifera*). The determination of dry matter, ash, protein, crude fat, dietary fiber, and digestible carbohydrates was performed. Moreover, the concentration of biogenic amines, low-molecular-weight carbohydrates, and mineral components, as well as fatty acid profile was determined. After the in vitro digestion, the in vitro bioaccessibility of the mineral components was established. The results of this study may contribute to the selection of the most effective hydrothermal treatment of Brussels sprouts in terms of the retention of analyzed nutrients and non-nutritive compounds.

## 2. Materials and Methods

### 2.1. Experimental Material

The analysis was performed on Brussels sprouts (*Brassica oleracea* var. *gemmifera*) purchased from a local supermarket (Cracow, Poland) in 2019. A batch of Brussels sprouts (2 kg raw weight, in triplicate) was pre-cleaned and divided into 4 sub-samples. Three sub-samples were subjected to the hydrothermal treatments: traditional cooking in water (boiling) (*n* = 3), steaming (*n* = 3), and sous vide (*n* = 3), and one remained raw, i.e., not subjected to any thermal treatment (*n* = 3). Parameters (time, temperature, and water ratio) of the hydrothermal treatment were optimized in previous studies based on the color and sensorial analyses, dry matter, protein, fat, ash, total dietary fiber, and soluble fraction content [4,5]. All hydrothermal treatments were carried until consumable softness was reached (tested using texturometer EZ Test X, Schimadzu’s, Hertogenbosch, The Netherlands). Conventional cooking in water was performed in stainless steel dishes using an induction hob (Hendi, Hamburg, Germany) for a period of 15 min at 98 ± 1 °C. The raw material-to-water ratio of 1:3 (m:V) was maintained (by carrying out the boiling process under cover to eliminate water evaporation). Steaming was conducted using a Retigo Orange Version 6 × GN1/1|O 611 in combi steamer (Retigo, Rožnov pod Radhoštěm, Czech Republic) for 7 min at 100 °C. Sous vide cooking was performed after vacuum packing (using vacuum packer VBN-4, RM Gastro, Veselí nad Lužnicí, Czech Republic) at 90 °C for about 45–50 min, using the Sous vide 225 448 system (Hendi, Hamburg, Germany). After completion of this part of the experiment, all samples were frozen at −20 ± 2 °C (blast chilling chamber, RedFox SHS-511, RM Gastro, Veselí nad Lužnicí, Czech Republic). After freezing, the vegetables were immediately lyophilized with a Christ Alpha 1-4 lyophilizer (Martin Christ Gefriertrocknungsanlagen GmbH, Osterode am Harz, Germany) and ground in a Tecator Knifetec 1095 laboratory mill (Foss Tecator, Uppsala, Sweden) to obtain a homogeneous material, which was stored under refrigeration in hermetically sealed containers. The material thus prepared was used for chemical analyses. In vitro digestion was performed on raw and thermally processed plant material (not subjected to freeze-drying).

### 2.2. Methods

#### 2.2.1. In Vitro Digestion

In vitro digestion was carried based on the methodology presented by other authors [15] with a modification [16]. Briefly, 0.5 g of homogenized plant material was placed in syringes, and distilled water was added and mixed. Then, 0.5 M HCl was acidified to pH = 2, and pepsin solution (concentration 6 mg/mL; activity 3850 U/mg; Sigma (P6887); dissolved in 0.1 M HCl) was added. Redistilled water was added, and the obtained solution was incubated at 37 °C for 2 h (in vitro digestion gastric fragment). After that, 1 M NaHCO_3_ was added in a volume providing pH = 7 and bile and pancreatin solution (bile and pancreatin concentrations of 90 mg/mL and 9 mg/mL, respectively; bile (Sigma-Aldrich B8631, St. Louis, MO, USA) dissolved in 0.1 M NaHCO_3_ and pancreatin (Sigma-Aldrich P7545; specific activity: 4 × USP specifications). Redistilled water was then added again (in vitro digestion intestinal fragment). Obtained solutions of the syringe were mixed and moved to dialysis bags (Sigma-Aldrich D9777-100FT, dialysis tubing cellulose membrane). They were closed and put into the imidazole buffer solution (3.4 g of imidazole (Sigma-Aldrich 792527) dissolved in 250 mL of redistilled water, pH adjusted to pH 7.0; supplemented with 11.688 g of NaCl anhydrous and redistilled water to 2 dm^3^). The flasks were then placed in a shaking water bath (40 rpm, GFL 1092) and incubated for 2 h at 37 °C. The obtained dialysates were stored at −22 ± 1 °C in tightly closed, airtight containers. They were used for further analyses (without freeze-drying, in the liquid form).

#### 2.2.2. Determination of Macronutrients Content

Dry matter content was determined by the dry-weighing method according to PN-ISO 712:2002 [17] with the use of a Venticell 55Plus dryer (BMT, Brno-střed, Czech Republic). Protein content was measured by the Dumas method according to PN-EN ISO 16634-1:2008 [18] using a TruSpec N device (LECO, Dallas, TX, USA). A coefficient of 5.9 was used to convert the determined nitrogen content into protein content. The ash content was calculated according to PN-EN ISO 2171:2010 [19] using a muffle furnace (model LE6/11/B150, Nabertherm, Lilienthal, Germany). Dietary fiber content was determined using the enzymatic–gravimetric method AOAC 991.43 (Megazyme kit, Megazyme, Lansing, MI, USA). The correctness of the method was verified using TDF Controls KIT (Megazyme, Lansing, MI, USA). Fat content was determined according to an intralaboratory validated test procedure, using the extraction and weighing method. Briefly, the fat was determined using the Leco TFE 2000 Analyzer by the supercritical CO_2_ extraction method. One gram of samples and one cubic centimeter of 2-propanol were weighed successively into the thimbles with diatomite. The thimbles were sealed with stoppers and placed in the analyzer. The assay was carried out at a temperature of 100 °C and a pressure of 9000 psi (LOQ = 0.1 g/100 g f.m.). The concentration of digestible carbohydrates was determined mathematically according to the formula:

Digestible carbohydrates content=100−(sum of ash, protein, fat and fiber in the sample)

#### 2.2.3. Determination of Mineral Components Content

The analysis of potassium, calcium, and magnesium content was performed based on the Polish standard PN-EN-15505:2009 [20]. The concentration of zinc, iron, manganese, and copper in samples was determined using the Polish Standard PN-EN 14084:2004 [21]. Mineral components were determined using the Atomic Absorption Spectrometry with the atomization in the flame FAAS method (Varian AA240FS of the Varian company, Dallas, TX, USA). The samples were previously mineralized with 65% nitric acid (Suprapur, MERCK, Kenilworth, NJ, USA, cat. no. 1.00441) using a high-pressure microwave method (MarsXPres, CEM, Chengdu, China). For calcium and magnesium analyses, Schinkel lanthanum chloride and cesium chloride buffer solutions of 10 g/L (MERCK, cat. no1.16755) were used. Schuhknecht and Schinkel buffers-aluminum nitrate and cesium chlorate at concentrations of 250 g/L and 50 g/L (MERCK, cat. no 102037) were used for the potassium analysis.

#### 2.2.4. Determination of Low-Molecular-Weight Carbohydrates (Glucose, Fructose, and Sucrose) Content

Prior to the analysis, samples were prepared according to the methodology presented by other authors [22]. Briefly, 20 g of each sample (raw and thermally processed Brussels sprouts) was homogenized with 60 mL of distilled water. Then, 1 mL arabinose (5 g/L) was added as standard to 10 mL of homogenate. Then, ethanol was added to a total volume of 50% (*v*/*v*) and extraction was carried out at room temperature for 30 min, periodically mixing the samples using vortex. Then, the cell material was centrifuged (2200× *g*, 15 °C, 10 min), and the supernatant was collected. Ethanol was added to a final concentration of 80% (*v*/*v*), and the samples were left for 30 min incubation at −20 °C in order to separate the protein precipitate. The samples were then centrifuged (2200× *g*, 2 °C, 15 min). The supernatant was collected, the ethanol was evaporated, and the residue was dissolved and filtered (0.45 µm). The solution thus obtained was used for glucose, fructose, and sucrose determinations.

To analyze the content of glucose, sucrose, and fructose (using the external calibration method prepared through the dilutions of the stock standard of D-glucose, D-sucrose, and D-fructose (from Sigma-Aldrich, Steinheim am Albuch, Germany) within the range of 50–100 μg/mL), the UltiMate 3000 Liquid Chromatography System was used. The chromatograph was composed of Autosampler (WPS-3000SL), Column Compartment with thermostat (3000SL), Gradient Pump (LPG-3400A), and Universal Chromatography Interface (UCI-50), all purchased from Dionex-Thermo Corp, (Sunnyvalle, CA, USA). Moreover, the refractive index detector, purchased from Knauer (Berlin, Germany) model 2400i, was set with the following parameters: signal intensity—RI@16x; time constant—2 s; thermostatization—85 °C. RI@16x was used throughout the study. The column used for analyses was the HPX87C column (300 × 7.8 mm, 8% cross linkage, 9 μm particle size) from Aminex (Dublin, Irleand) in isocratic mode. According to Aminex bulletin 1895 with the use of ultra-pure deionized water. Chromeleon 6.80 software was used for data collection and processing.

#### 2.2.5. Determination of Fatty Acids Profile

Fat extraction was performed with liquid carbon dioxide at a purity level of 4.5 using TFE 2000 (Leco, Dallas, TX, USA). The extracted fat was esterified by the method of Ledoux et al. [23]. For this purpose, the obtained fraction was dissolved in 50 µL of toluene, and 100 µL of 2M sodium methoxide was added. The esterification was carried out at room temperature for 20 min. Then, 0.5 cm^3^ of a 14% methanolic solution of boron trifluoride was added, and it was left at room temperature for 20 min. The fatty acid methyl esters were extracted twice with 2 cm^3^ of hexane.

Fatty acids were analyzed by gas chromatography using a TRACE 1300 gas chromatograph (Thermo Fisher Scientific S. p. A, Milano, Italy) with a flame ionization detector (FID), equipped with a ZB-FAME column (60 mm × 0.25 mm × 0.20 mm). The carrier gas was helium (5 mL/min). Split flow—10 mL/min. Split ratio—3:1. Detector temperature FID −240 °C, Injector temperature −220 °C. Column temperature—from 60 °C (holding for 3 min) to 200 °C with an increment of 7 °C/min and kept at 200 °C for 20 min. Fatty acids were identified by comparing the retention times of fatty acid methyl esters (FAME) to a Supelco 37 FAME Mix (Sigma-Aldich Co., St. Louis, MO, USA). The fatty acid profile was determined by identifying and calculating the relative peak areas.

#### 2.2.6. Determination of Biogenic Amines Content

Biogenic amines determination was carried using a Dionex Ultimate 3000 HPLC apparatus (Thermo Scientific, Waltham, MA, USA) with the four-channel fluorescent detector FLD 3400RS (Thermo Scientific) together with the low-pressure gradient pump containing a four channel mixer. The emission and excitation wavelengths were 540 nm and 340 nm, respectively (flow cell temperature: 30 °C). A Kromasil 100-5-C18 4.6 × 250 mm column (Akzo Nobel, Amsterdam, The Netherlands) at aa 30 °C column temperature was used for separation. The flow rate was set at 0.8 mL/min with two mobile phases: (A) acetonitrile (Merck, Darmstadt, Germany) and (B) HPLC grad water (Merck, Germany). There were six steps of the elution program: (I) 65% A and 35% B for 1 min, (II) increasing to 80% A for 9 min, (III) increasing to 90% A for 2 min, (IV) increasing to 95% A for 4 min, (V) holding for 7 min, and (VI) back to 65% A and holding for 5 min. Chromeleon 7.0 software was used to collect and calculate data. The solution of standards of eight biogenic amines was prepared in 0.1 M HCl at a concentration of 1 mg/cm^3^ for every amine. The following standards used were: tyramine hydrochloride, tryptamine hydrochloride, histamine dihydrochloride, putrescine dihydrochloride, cadaverine dihydrochloride, spermine tetrahydrochloride, spermidine trihydrochloride, and 2-phenylethylamine hydrochloride. All standards solutions and all reagents used were of HPLC grade (Sigma-Aldrich, Switzerland). The limit of detection (LOD) was 0.0002 μg/cm^3^, while the limit of quantification (LOQ) was 0.001 mg/kg. The R2 = 0.998. A three-point calibration using 1 µg/mL, 0.5 µg mL, and 0.25 µg mL standards was used.

### 2.3. Bioaccessibility

The bioaccessibility of the mineral components was calculated according to the following formula [24]:

$$\mathrm{bioaccessibility}\;(\%)=\frac{\mathrm{bioaccessible}\;\mathrm{amount}\;\mathrm{of}\;\mathrm{the}\;\mathrm{component}\;\mathrm{from}\;\mathrm{vegetable}\;\mathrm{sample}\;(\mathrm{after}\;\mathrm{in}\;\mathrm{vitro}\;\mathrm{digestion})}{\mathrm{total}\;\mathrm{amount}\;\mathrm{of}\;\mathrm{the}\;\mathrm{component}\;\mathrm{from}\;\mathrm{vegetable}\;\mathrm{sample}\;(\mathrm{before}\;\mathrm{in}\;\mathrm{vitro}\;\mathrm{digestion})}\times 100\%$$

### 2.4. Statistical Analysis

The analyses and heat treatments were performed in triplicate. The results were expressed as mean value ± standard deviation. The results were statistically processed using one-way analysis of variance (ANOVA). The significance of the differences between the mean values was determined by Duncan’s post hoc test or by Tukey’s HSD post hoc test at a significance level of *p* ≤ 0.05. The Statistica 13 software package (StatSoft Inc., Tulsa, OK, USA) was used for the calculations.

## 3. Results and Discussion

### 3.1. Analysis of Macronutirents Content in Brussels Sprouts Raw and Subjected to Hydrothemal Treatments

The concentration of individual macronutrients in raw and cooked Brussels sprouts is shown in Table 1. The type of heat treatment applied to Brussels sprouts (*Brassica oleracea* var. *gemmifera*) had a larger effect on the dry matter content compared to the raw sample only in the case of traditional cooking in water (decrease in dry matter concentration due to leaching of components into the water medium) (*p* ≤ 0.05). Sous vide, due to prior vacuum packaging of the product, reduces the elution of soluble compounds into the aqueous medium, while steaming is connected the evaporation of water from the product, thus influencing its mass and the dry to fresh mass ratio. These correlations probably influenced the preservation of dry matter at a level comparable with raw Brussels sprouts (*p* ≤ 0.05). Other authors observed significant changes in the dry matter content of *Brassica oleracea* var. *gemmifera* also during traditional cooking in water (decrease) but the same trend was also observed during sous vide processing, with, as in the case of our study, no change in the case of steam cooking [4,6].

Similarly to our own study, other authors have also demonstrated that the concentration of dry matter of previously blanched and frozen swede rods (*Brassica napus* var. *napobrassica*) subjected to preheated sous vide and boil-in-bag treatment was statistically higher than conventional cooking in water [7]. Other authors reported that a divergent dependence of a decrease in dry matter concentration for all thermal treatments (boiling in water, steaming, and microwaving) of broccoli (*Brassica oleracea* var. *italica*) and cauliflower (*Brassica oleracea* var. *botrytis*) occurred compared to the raw sample [8]. The dissimilarity in the results of dry matter concentration may be caused by the difference in vegetable species, their morphological structure, growing conditions, and harvesting date. Moreover, it can also result from the differences in the conditions of the hydrothermal processes conducted (processing time and temperature).

The heat treatment of *Brassica oleracea* var. *gemmifera* by traditional cooking in water was associated with the lowest total ash concentration by leaching of mineral components into water. The use of sous vide and steam methods had no effect on the ash content of Brussels sprouts. This result correlates with the concentration of individual mineral components also presented in this article, in which the sous vide method and steaming were mostly characterized by the preservation of mineral components at a level comparable to the raw sample. This relationship is possibly due to the preservation of mineral components by due to the reduced direct contact of the raw material with the aqueous medium. Additionally, other authors showed a similar ash content for white rose cauliflower and steamed broccoli cooked sous vide compared to the raw sample [4]. Contrary to the results obtained, other researchers showed a significant reduction in total ash content for all hydrothermal treatments used (boiling in water, steaming, microwaving) compared to raw material of cauliflower and broccoli [8,9].

The heat treatment of Brussels sprouts had an impact on protein concentrations in this vegetable. Using the sous vide method allowed the highest protein retention compared to the raw sample, while boiling contributed to the highest loss of this component. Similar protein concentrations for steaming and pressure cooking of mustard leaves (*Brassica juncea*) in comparison to the raw sample were also established [10]. Additionally, in the case of steam cooking, steam blanching, and microwaving of white rose cauliflower, a similar value of this component was found to that of the raw material not subjected to hydrothermal treatment [9]. Other researchers, also as in our own study, showed the highest losses of this component with traditional cooking in water, indicating at the same time that the protein losses during thermal treatment are related to the process of its denaturation as a result of the application of high temperatures. This indicates that the highest loss of this component in boiling may be related to the partial leaching of water-soluble protein compounds from the material [4,8,9]. Considering the results, the highest protein retention is therefore justified for the samples subjected to the sous vide method, as this thermal treatment method, due to the vacuum packaging of the product, prevents direct contact with the medium and has the lowest processing temperature of all the hydrothermal treatments used.

Steaming and boiling were associated with a significant reduction in the fat content of Brussels sprouts, directly linked to the process of fat oxidation at high temperatures and its possible loss through leakage into the water in the case of traditional cooking in water. In contrast, the sous vide method had no effect on the concentration of this component with respect to the raw material, allowing the highest level of fat retention in *Brassica oleracea* var. *gemmifera*. A similar trend of decreased fat content in white rose cauliflower and broccoli following microwaving, steaming, and traditional cooking in water was also reported by other authors [8].

Boiling had a reducing effect on fiber concentration in *Brassica oleracea* var. *gemmifera*; as for other components, it was associated with the highest percentage loss compared to the uncooked raw material. To a lesser extent than conventional cooking, sous vide also led to a reduction in fiber concentration. Conversely, steaming Brussels sprouts allowed the best level of preservation of this component at a statistical level as the raw sample. A decrease in fiber concentration was seen as a result of all heat treatments (boiling in water, steaming, and microwaving) of broccoli and cauliflower [8]. Meanwhile, no effect of heat treatment on the fiber content of cauliflower except its increase as a result of boiling in water was established by other authors, indicating that heat treatment affects not only the solubility but also the physicochemical properties of fiber [9].

All the hydrothermal treatments applied to Brussels sprouts resulted in a reduction in the concentration of available carbohydrates, with the lowest concentration in the case of traditional cooking in water by leaching from the product. Sous vide and steaming had equal influence on the concentrations of digestible carbohydrates in *Brassica oleracea* var. *gemmifera*. A similar correlation was observed in the case of broccoli and cauliflower in one study [8], while conversely, oppositely in another study, besides the preparation of cauliflower by stir-frying, authors did not show any effect of other heat treatments on the concentration of digestible carbohydrates [9].

Based on these results, it can be concluded that the sous vide technique, due to the use of membrane heating (through prior vacuum packaging), which prevents direct contact of the raw material with the water medium and simultaneously a lower range of applied temperatures, remains a better method of vegetable cooking than boiling in the case of nutritional value of product. Furthermore, it can also be an alternative to preparing vegetables by steaming.

### 3.2. Analysis of Fatty Acid (FA) Profile of Brussels Sprouts Raw and Subjected to Hydrothemal Treatments

The fatty acid profile of raw and hydrothermally treated Brussels sprouts is presented in Table 2. Thirteen fatty acids were identified in all Brussels sprouts samples, and only in the case of Brussels sprouts cooked traditionally in water was the presence of heptadecanoic acid additionally demonstrated. For all analyzed samples, the highest percentage of fatty acids was represented by α-linolenic acid (50.90–56.84% of total FA), linolenic acid (17.98–19.60% of total FA), and palmitic acid (14.65–16.71% of total FA). The rest of the fatty acids analyzed were present in contents not exceeding 4% of total FA. Raw Brussels sprouts had lower or comparable percentage levels of individual fatty acids to rape Brussels sprouts (*Brassica oleraceae* var *costata*) and ‘tronchunda’ cabbage (*Brassica napus* var *napus*), however with slight differences in percentage share of PUFA and SFA [25]. The analyzed *Brassica oleracea* var. *gemmifera* had a different fatty acid profile from three broccoli crops (Baeridom, AMaGi, and Cheonjae) and cauliflower (Asia white, Asia purple and Bridal), with variation mostly within percentage share of PUFA and SFA [26]. These differences may be due to both the different species of *Brassica* vegetables analyzed and the discrepancy in the method of cultivation and the different country of origin.

Hydrothermal treatment had an effect on the fatty acid profile compared to the raw sample for palmitic, palmitoleic, margaroleic, stearic, oleic, vaccenic, α-linolenic, *cis*-11,14-eicosadienoic, and docosahexaenoic acid. The results showed that boiling contributed mostly to the largest proportion of PUFA in the analyzed Brussels sprouts. Sous vide turned out to be the best method of hydrothermal treatment of *Brassica oleracea* var. *gemmifera* in relation to the percentage share of MUFA. Meanwhile, steaming as a hydrothermal treatment led to the retention of the largest proportion of SFA. In addition, it should be noted that in all selected hydrothermal treatment methods, as well as in the unprocessed raw material, the largest proportion of fatty acids included in the profile of *Brassica oleracea* var. *gemmifera* was unsaturated fatty acids (more than 70%), which may indicate the health-promoting properties of this vegetable.

### 3.3. Analysis of Biogenic Amines Concentration of Brussels Sprouts Raw and Subjected to Hydrothemal Treatments

Table 3 shows the concentration of biogenic amines in raw Brussels sprouts and those subjected to three hydrothermal treatments. Spermidine (16.68 ± 1.04 mg/kg d.m.) and histamine (14.21 ± 0.91 mg/kg d.m.) were the main biogenic amines in raw *Brassica oleracea* var. *gemmifera*. In contrast, tryptamine (0.31 ± 0.01 mg/kg d.m.) and cadaverine (0.01 ± 0.00 mg/kg d.m.) were detected in the lowest concentrations. Another study, like our study, indicated spermidine as the biogenic amine with the highest concentration in raw white cabbage (*Brassica oleracea* L. var. *Capitata* cv. *Bronco*), while histamine was conversely the biogenic amine present in the lowest content [27].

Low concentrations of biogenic amines such as those obtained in our study are a desirable result because biogenic amines in low concentrations are necessary for normal metabolic processes and cell growth and differentiation, while in high concentrations they may develop toxic effects [28].

Heat treatment had an effect on the concentration of individual biogenic amines in Brussels sprouts. In the case of putrescine, cadaverine, and histamine, all hydrothermal treatments contributed to a reduction in the content of these biogenic amines, reaching the lowest levels in boiled (putrescine and histamine) and sous vide (cadaverine) Brussels sprouts. Traditional cooking in water as the only heat treatment method for Brussels sprouts contributed to a significant increase in tyramine and spermine concentration. Spermidine levels decreased in sous vide Brussels sprouts, in contrast to the increase due to steaming and boiling. An inverse correlation was observed for 2-phenylethylalanine. The concentration of tryptamine, as the only biogenic amine, was increased by each heat treatment method compared to raw Brussels sprouts. In most cases, traditional cooking in water allowed for lower retention of biogenic amines than sous vide and steaming, with the exception of tryptamine, tyramine, spermidine, and spermine. The mainly reducing effect of heat treatment on biogenic amines is a desirable result due to their toxic effects at high concentrations (levels that are dangerous for health are histamine at 200–400 mg/kg; tyramine at level 125 mg/kg; for other amines, concentration over 1000 mg/kg is inadvisable). These results are in opposition to those obtained by fermentation of Brussels sprouts and other *Brassica* vegetables, due to the activity of microorganisms contributing to an increase in the concentration of biogenic amines [27,29,30].

### 3.4. Analysis of Mineral Components Concentration of Brussels Sprouts That Were Raw or Subjected to Hydrothemal Treatments before and after the In Vitro Digestion; Determination of In Vitro Bioaccessibility of the Mineral Components

The concentrations of potassium, sodium, calcium, magnesium, manganese, iron, and zinc were determined in the analyzed samples. Their values are presented in Table 4. The mineral component that was present in the highest concentration in raw Brussels sprouts was potassium, while manganese had the lowest amount among the mineral components analyzed. These results are in agreement with those obtained by other authors, who also in the case of Brussels sprouts showed the highest concentration of potassium and the lowest of copper and manganese [6].

An effect of heat treatment on the content of all minerals except iron and magnesium was demonstrated. Conventional cooking in water led to the highest losses of mineral constituents through leaching of the ingredients into the medium, thus showing that this heat treatment method for Brussels sprouts is the least effective in terms of potassium, sodium, calcium, manganese, and zinc retention. A similar trend was observed in another study, indicating that boiling is the method with the highest percentage reduction in mineral concentrations in white rose cauliflower by leaching during the process into the aqueous medium. Additionally, that study emphasized that steam-boiling, steam-blanching, stir-frying, and microwaving of *Brassica oleracea* var. *botrytis L.* enable a more efficient retention of minerals, which matches the results of our own study [9]. Similarly, in line with our own results, other authors indicated that steam boiling is a more effective thermal treatment method for broccoli and cauliflower regarding mineral retention compared to boiling in water and using microwaves [8]. Additionally, in our study, steaming and sous vide were better methods for hydrothermal treatment of *Brassica oleracea* var. *gemmifera* with respect to mineral preservation, as a result of lack of elution of mineral components into the medium. However, sous vide-treated Brussels sprouts showed higher levels of minerals than the steamed sample. It is also worth noting that, in the case of potassium, sodium, magnesium, iron, and zinc, the sous vide method enabled these minerals to be preserved at a consistent level, as was the case in raw Brussels sprouts, thus representing, among the three hydrothermal processing methods used, the best choice for preserving the mineral values of the product. Our own results are in agreement with those obtained in another study, in which the authors also indicated that sous vide is the processing method for *Brassica* vegetables (Brussels sprouts, broccoli, and white and green rose cauliflower) that allows maximum retention of mineral components compared to steaming and traditional cooking in water [6]. Manganese in vegetables occurs mainly in the form of water-insoluble compounds. As a result of the hydrothermal treatment, the ratio of dry weight to fresh weight of the vegetable changes, and, moreover, some water-soluble compounds are lost. Thus, the manganese content of the raw vegetable may be lower than that of the cooked vegetables, which results from the differences in the above-mentioned ratio of dry and fresh weight between raw and thermally treated vegetables.

Table 5 shows the results of mineral concentrations in the samples after in vitro digestion. Compared to the samples not subjected to in vitro digestion (Table 4), the content of individual minerals decreased significantly. Manganese and iron in the dialysates were present in concentrations lower than the LOQ, while sodium, due to the use of NaHCO_3_ during the in vitro procedure, was omitted from the results due to its interference with the sodium concentration in the reagent. The bioaccessibility of minerals from plant dietary sources is directly related to the type of processing of the plant raw material, the chemical form of the mineral, and the presence of components that reduce bioaccessibility (such as tannins, phytates, and fiber) and enhance bioaccessibility (such as amino acids) [31].

In the case of magnesium, the type of heat treatment had no effect on the content of this compound in digested samples, and the same trend was observed for raw materials before digestion. The highest bioaccessibility of magnesium was found in sous vided and boiled Brussels sprouts (5.04 ± 0.16% and 5.02 ± 0.20%, respectively) and the lowest in raw Brussels sprouts (4.54 ± 0.08%). Additionally, for calcium, a trend similar to the raw material before digestion was observed; after in vitro digestion, the sous vide hydrothermal treatment of Brussels sprouts allowed statistically similar levels of calcium compared to the raw sample, while conventional cooking in water and steaming were associated with higher losses of this component, with a simultaneous lowest bioaccessibility for the raw sample (6.85 ± 0.43%) and highest for boiling (10.17 ± 0.65%). The highest bioavailability of calcium and magnesium in boiled Brussels sprouts is directly related to the highest loss of bioaccessibility-limiting components such as phytates and fiber by traditional cooking (Table 1). This is also in agreement with the lowest bioaccessibility of magnesium and calcium in raw *Brassica oleracea* var. *gemmifera*, in which the concentration of components limiting the bioaccessibility of mineral components is the highest [31]. The type of heat treatment chosen also had an effect on the concentration of zinc in plant material digested in vitro, with the lowest content of this component in the case of Brussels sprouts boiled in water and a similar value for steamed Brussels sprouts. *Brassica oleracea* var. *gemmifera* treated by sous vide preserved the highest level of zinc among the hydrothermal treatments used at a level similar to the plant material. In parallel, the bioaccessibility was lowest for cooked Brussels sprouts (6.57 ± 0.06%) and highest for raw ones (17.02 ± 1.37%). The discrepancy in bioaccessibility of zinc in relation to calcium and magnesium may be due to the lowest ratio of reduction of zinc concentration after in vitro digestion in relation to the plant material before this procedure in opposition to other minerals. Similarly to the case of zinc, the trend of the highest mineral concentration in the raw sample after in vitro digestion was observed for potassium. Its content successively decreased for steamed and sous vide Brussels sprouts and reached the lowest value in the case of traditional cooking in water, which as a method of hydrothermal treatment, similarly to the sample before digestion, caused the highest loss of this component. Prior to in vitro digestion, there was a high degree of potassium leaching from the product into the medium during boiling. The percentages of potassium bioaccessibility were highest for raw *Brassica oleracea* var. *gemmifera* (6.10 ± 0.01%) and lowest for Brussels sprouts cooked traditionally in water (5.17 ± 0.07%). Due to the solubility of monovalent cations such as K+ in opposition to divalent cations across the pH spectrum, their interaction with phytic acid was less limiting in terms of bioaccessibility than in the case of divalent cations. Thus, in the case of potassium, the effect of hydrothermal treatment on the bioaccessibility of this component is lower than that for other minerals [31]. The concentration of iron, manganese, copper, and zinc in leafy vegetables (lettuce, edible amaranth, leaf lettuce, swamp cabbage, cole, pak choi, and spinach) and root vegetables (white radish, carrot, potato) after in vitro digestion was also lower than in the plant material before digestion, as in our study. Zinc had a similar bioaccessibility range as in the analyzed Brussels sprouts, while in opposition to our own study, the authors also detected the presence of iron and manganese in leafy and root vegetables after digestion. The discrepancies in the bioaccessibility of individual minerals may be directly due to the difference in species of the analyzed products, the divergent methodologies of the in vitro digestion used, and the sensitivity of the mineral determination [19].

### 3.5. Analysis of Low-Molecular-Weight Carbohydrates Concentration of Raw Brussels Sprouts and Those Subjected to Hydrothemal Treatments before and after In Vitro Digestion

The glucose concentration in raw and heat-treated Brussels sprouts before and after in vitro digestion is shown in Table 6. Hydrothermal treatments had no effect on glucose concentration in *Brassica oleracea* var. *gemmifera* compared to raw material. The glucose concentration in raw Brussels sprouts (170.58 ± 13.83 mg/g dry matter) was lower than that found in raw white cabbage (254 ± 36 mg/g dry matter) in other research but comparable to the level found by other authors in cauliflower (140 ± 40 mg/g dry matter) and higher than in kale [24]. Lower glucose concentrations than those found in our study were also detected in raw broccoli and cauliflower in another study [26]. The differences in the case of glucose concentrations may depend both on the vegetable species, cultivation methods, harvest date, and country of origin. The choice of heat-treatment method had no effect on glucose concentration in the plant material studied. A different correlation to our study—i.e., a decrease in glucose concentration in *Brassica oleracea* var. *capitata* after heat treatment (blanching) relative to the raw vegetable—was observed in other studies on the sugar profile in white cabbage [32]. The glucose concentration decreased after in vitro digestion relative to the vegetable before digestion. Following in vitro digestion, no differences between raw and thermally treated Brussels sprouts were observed. As in the case of plant material’s prior digestion, the choice of heat treatment method had no effect on glucose concentration in the dialysates. The concentrations of bioaccessible glucose in examined Brussels sprouts were higher than those in cooked pulp of sweet potato, comparable to those in winter squash, and lower than those in raw sweet potatoes in the results of other authors. These differences may result from different vegetables species and dissimilarities in in vitro digestion procedures [33,34]. In comparison to values of glucose digestibility in beet and cane during fermentation, the level of glucose retention was lower in thermally processed Brussels sprouts than in the case of fermented molasses. This may be mainly due to the different processing type, initial glucose concentration, and dissimilarities in in vitro digestion procedure [35].

The sucrose concentration in raw and heat-treated Brussels sprouts before and after in vitro digestion is shown in Table 6. Sucrose level in raw Brussels sprouts (303.74 ± 5.41 mg/g dry matter) was higher than that in raw white cabbage, cauliflower, and kale found by other authors (range of 1.7–61 mg/g dry matter) [22]. Lower sucrose concentrations were also detected in raw broccoli and cauliflower in another study compared to our study [26]. Differences may result from species, country of origin, and harvesting time. As was the case with glucose, hydrothermal treatments also had an effect on sucrose concentration in *Brassica oleracea* var. *gemmifera* in comparison to raw material, with the exception of sous vide technique, probably due to the longer treatment time. There were no differences between sucrose concentration depending on the type of thermal treatment applied. The inverse relationship of the decrease in sucrose concentration as a result of blanching was found by other authors in the case of cabbage [32]. This difference may result from shorter processing time (blanching for 5 min) than the ones applied in this study and also from a different species of the analyzed *Brassica* vegetable. The sucrose concentration in the vegetable after in vitro digestion compared to before digestion changed only in the case of boiling (in which case it increased). Sucrose digestibility in beet and cane during fermentation was lower than the retention level of sucrose in *Brassica oleracea* var. *gemmifera*. This may be due to dissimilarity in the initial sucrose concentration in the plant raw material and the treatment method used [35].

Fructose concentration in raw and thermally treated *Brassica oleracea* var. *gemmifera* is presented in Table 6. Fructose concentration in raw Brussels sprouts (86.24 ± 1.75 mg/g dry matter) was in the same range determined by other authors in cauliflower, kale, and cabbage (58–220 mg/g dry matter) [22]. Meanwhile, another study found higher fructose concentrations than our study in raw broccoli and cauliflower, except for florets of AMaGi broccoli [26]. Depending on the species, growing conditions and harvest time, such differences can occur. As was the case for sucrose and glucose, fructose concentration also did not change for the most part after thermal treatments with regard to raw material, except with the sous vide technique. The type of hydrothermal treatment applied had no influence on fructose concentration (except for the sous vide technique). The observed dependence of no changes in fructose concentration as a result of thermal treatment compared to the raw sample is in opposition to the relation presented by other authors as a result of cabbage blanching [32]. The fructose concentration decreased after in vitro digestion in comparison to the vegetable before digestion. Following in vitro digestion, no differences between raw and thermally treated Brussels sprouts were observed. As was the case for glucose, fructose digestibility in beet and cane during fermentation was also higher than in case of *Brassica oleracea* var. *gemmifera*. This indicates a difference in the influence of the processing type (fermentation and hydrothermal treatment) on concentration of fructose [35]. Lower concentrations of fructose in rice straw were found in another study compared to in our study; however the decreasing tendency after processing (ensiling of rice straw) was similar to our findings. Differences may result from initial fructose concentration, different processing type and dissimilarities in in vitro digestion procedure [36].

## 4. Conclusions

The type of heat treatment chosen has a significant effect on the concentration of selected nutrients and non-nutrients and in vitro bioaccessibility of minerals in *Brassica oleracea* var. *gemmifera*. The leaching of nutrients and non-nutrients to an aqueous medium during boiling involves the highest percentage losses. Steaming and sous vide as methods of cooking Brussels sprouts allow for a higher level of preservation of the individual compounds. By using reduced processing temperatures and vacuum packaging, sous vide cooking can be used as an alternative to traditional cooking to preserve the higher nutritional value of *Brassica oleracea* var. *gemmifera*. The presented research results focus only on the concentration of the selected nutrients and non-nutritive compounds of Brussels sprouts that are raw and those thermally treated and subjected to in vitro digestion. Further analyses should concentrate on the content of individual bioactive compounds and antioxidant activity of *Brassica oleracea* var. *gemmifera* that are raw and those thermally treated and subjected to in vitro digestion in order to determine the effect of hydrothermal treatment on the bioactive potential of Brussels sprouts.

## Figures and Tables

**Table 1:** Dry matter content and the chemical composition of Brussels sprouts raw and after thermal treatments (g/100 g f.m.).

Treatment		Dry Matter	Ash	Protein	Crude Fat	Dietary Fiber	Digestible Carbohydrates
Raw		15.93 ± 0.42 b	1.13 ± 0.02 b	3.45 ± 0.01 d	0.30 ± 0.02 b	5.26 ± 0.04 c	5.76 ± 0.04 c
Thermal treatments	Steaming	15.05 ± 0.73 b	1.10 ± 0.01 b	3.29 ± 0.00 b	0.20 ± 0.02 a	5.27 ± 0.06 c	5.20 ± 0.00 b
	Sous vide	15.16 ± 0.29 b	1.13 ± 0.02 b	3.43 ± 0.01 c	0.26 ± 0.01 b	5.11 ± 0.06 b	5.18 ± 0.00 b
	Boiling	14.05 ± 0.20 a	0.98 ± 0.00 a	3.09 ± 0.01 a	0.17 ± 0.01 a	4.76 ± 0.07 a	5.05 ± 0.01 a

Results are shown as mean ± standard deviation (SD). Means in a column followed by the same letter (a,b,c,d) are not significantly different (*p* < 0.05); f.m. fresh matter.

**Table 2:** Fatty acid profile (values expressed as % of total fatty acids) of raw and thermally processed Brussels sprouts.

Fatty Acid	Raw	Thermal Treatment		
		Steaming	Sous Vide	Boiling
Myristic acid (C14:0)	0.21 ± 0.02 a	0.17 ± 0.04 a	0.20 ± 0.01 a	0.21 ± 0.02 a
Pentadecanoic acid (C15:0)	0.24 ± 0.06 a	0.28 ± 0.03 a	0.25 ± 0.00 a	0.24 ± 0.00 a
Palmitic acid (C16:0)	16.71 ± 0.20 c	15.88 ± 0.07 b	14.65 ± 0.03 a	14.94 ± 0.08 a
Heptadecanoic acid (C17:0)	<LOQ	<LOQ	<LOQ	0.22 ± 0.00
Stearic acid (C18:0)	3.95 ± 0.27 b	3.84 ± 0.13 b	2.58 ± 0.03 a	2.23 ± 0.05 a
Palmitoleic acid (C16:1)	0.98 ± 0.01 a	1.16 ± 0.01 b	1.41 ± 0.10 c	1.31 ± 0.06 bc
Margaroleic acid (C17:1)	1.03 ± 0.04 b	1.59 ± 0.05 c	1.47 ± 0.08 c	0.32 ± 0.02 a
Oleic acid (C18:1n9c)	1.33 ± 0.02 c	0.97 ± 0.12 b	1.05 ± 0.04 b	0.74 ± 0.00 a
Vaccenic acid (C18:1n7)	3.15 ± 0.03 ab	3.07 ± 0.15 a	3.51 ± 0.05 c	3.33 ± 0.00 bc
Linoleic acid (C18:2n6c)	19.11 ± 1.07 ab	19.60 ± 0.10 b	19.13 ± 0.06 ab	17.98 ± 0.07 a
α-Linolenic acid (C18:3n3)	50.90 ± 0.52 a	50.90 ± 0.16 a	51.90 ± 0.26 b	56.84 ± 0.14 c
cis-11.14-Eicosadienoic acid (C20:2)	0.29 ± 0.02 ab	0.24 ± 0.03 a	0.32 ± 0.00 b	0.26 ± 0.01 a
Arachidonic acid (C20:4n6)	0.53 ± 0.01 a	0.54 ± 0.10 a	0.52 ± 0.15 a	0.59 ± 0.05 a
Docosahexaenoic acid C22:6n3	1.57 ± 0.01 b	1.78 ± 0.12 c	3.00 ± 0.08 d	0.80 ± 0.00 a
SFA	21.11	20.17	17.68	17.84
MUFA	6.49	6.79	7.44	5.70
PUFA	72.4	73.06	74.87	76.47

Results are shown as mean ± standard deviation (SD); means followed by the same letter (a,b,c,d) in a row are not significantly different (*p* < 0.05); SFA—saturated fatty acids; PUFA—polyunsaturated fatty acids; MUFA—monounsaturated fatty acids; heptadecanoic acid LOQ = 0.05 mg/100 g f.m.

**Table 3:** Concentration of biogenic amines in raw and thermally treated Brussels sprouts (mg/kg d.m.).

Biogenic Amine	Raw	Thermal Treatment		
		Steaming	Sous Vide	Boiling
Tryptamine	0.31 ± 0.01 a	1.12 ± 0.01 d	0.73 ± 0.01 b	1.08 ± 0.01 c
2-Phenylethylalanine	0.12 ± 0.01 b	0.10 ± 0.02 b	0.17 ± 0.00 c	0.07 ± 0.00 a
Putrescine	3.56 ± 0.07 c	2.86 ± 0.06 b	2.67 ± 0.08 b	1.19 ± 0.02 a
Cadaverine	0.01 ± 0.00 c	0.01 ± 0.00 b	0.00 ± 0.00 a	0.00 ± 0.00 b
Histamine	14.21 ± 0.91 c	11.20 ± 0.50 b	13.58 ± 1.25 c	6.31 ± 0.19 a
Tyramine	5.50 ± 0.16 c	2.42 ± 0.19 b	1.56 ± 0.14 a	6.15 ± 0.30 d
Spermidine	16.68 ± 1.04 b	20.25 ± 0.81 c	9.89 ± 0.29 a	24.36 ± 0.34 d
Spermine	0.83 ± 0.13 c	0.46 ± 0.04 b	0.25 ± 0.04 a	1.46 ± 0.07 d

Results are shown as mean ± standard deviation (SD); means followed by the same letter (a,b,c,d) in a row are not significantly different (*p* < 0.05); d.m.—dry matter.

**Table 4:** Content of selected mineral components in raw and thermally treated Brussels sprouts (mg/kg d.m.).

Mineral Component	Raw	Thermal Treatment		
		Steaming	Sous Vide	Boiling
Potassium	26,327.15 ± 483.29 c	24,699.68 ± 618.87 b	25,991.36 ± 187.01 bc	21,899.37 ± 715.87 a
Sodium	647.47 ± 1.77 c	577.41 ± 10.98 b	653.89 ± 0.85 c	495.08 ± 5.66 a
Calcium	2219.22 ± 45.60 c	1512.06 ± 16.65 ab	1638.90 ± 76.32 b	1336.87 ± 99.65 a
Magnesium	1033.77 ± 80.54 a	992.50 ± 57.00 a	1034.91 ± 9.98 a	954.78 ± 32.26 a
Manganese	13.01 ± 0.61 a	13.09 ± 0.40 a	14.74 ± 0.09 b	14.30 ± 0.11 b
Iron	32.32 ± 3.04 a	34.65 ± 1.78 a	36.62 ± 2.15 a	32.85 ± 3.26 a
Zinc	21.47 ± 1.35 b	17.40 ± 0.33 a	20.52 ± 0.85 b	16.78 ± 0.09 a

Results are shown as mean ± standard deviation (SD); *n* = 3; means followed by the same letter (a,b,c) in a row are not significantly different (*p* < 0.05); d.m.—dry matter.

**Table 5:** Content of selected mineral components in raw and thermally treated Brussels sprouts after in vitro digestion (mg/kg d.m.).

Mineral Component		Raw	Thermal Treatment		
			Steaming	Sous Vide	Boiling
Potassium	A	1605.38 ± 31.82 c	1356.38 ± 45.66 b	1343.96 ± 8.38 b	1143.39 ± 2.28 a
	B	6.10 ± 0.01	5.49 ± 0.05	5.22 ± 0.16	5.17 ± 0.07
Calcium	A	151.9 ± 56.48 b	138.29 ± 1.18 a	149.52 ± 1.97 b	135.70 ± 1.49 a
	B	6.85 ± 0.43	9.15 ± 0.18	9.13 ± 0.30	10.17 ± 0.65
Magnesium	A	46.92 ± 4.50 a	48.55 ± 1.62 a	52.13 ± 2.19 a	47.94 ± 0.27 a
	B	4.54 ± 0.08	4.90 ± 0.44	5.04 ± 0.16	5.02 ± 0.20
Zinc	A	3.64 ± 0.06 c	1.19 ± 0.01 a	1.45 ± 0.07 b	1.10 ± 0.00 a
	B	17.02 ± 1.37	6.83± 0.20	7.06 ± 0.61	6.57 ± 0.06
Manganese		<LOQ	<LOQ	<LOQ	<LOQ
Iron		<LOQ	<LOQ	<LOQ	<LOQ

Results are shown as mean ± standard deviation (SD); *n* = 3; means followed by the same letter (a,b,c) in a row are not significantly different (*p* < 0.05); d.m. dry matter; A—values after in vitro digestion (mg/kg d.m.); B—percentage of bioaccessibility (%); LOQ values (for manganese LOQ = 0.2 mg/kg and iron LOQ = 0.6 mg/kg).

**Table 6:** Content of low molecular weight carbohydrates in Brussels sprouts that were raw and those subjected to hydrothermal treatments before and after in vitro digestion (mg/g d.m.).

Low-Molecular-Weight Carbohydrates	Sample Type (Variable a)	Raw	Thermal Treatment (Variable b)			Results of Two-Factorial ANOVA		
			Steaming	Sous Vide	Boiling	a	b	a*b
Glucose	A	170.58 ± 13.83 b	135.20 ± 10.39 b	152.81 ± 3.47 b	160.29 ± 0.18 b	0.00	0.01	0.88
	B	80.97 ± 0.13 a	52.08 ± 2.34 a	59.93 ± 0.37 a	69.73 ± 3.50 a			
Sucrose	A	303.74 ± 5.41 d	311.47 ± 3.68 bd	331.24 ± 5.45 c	306.86 ± 0.89 d	0.00	0.00	0.00
	B	300.87 ± 0.28 d	325.52 ± 3.20 bc	329.94 ± 1.12 c	352.99 ± 0.97 a			
Fructose	A	86.24 ± 1.75 b	85.56 ± 2.82 b	118.32 ± 5.30 a	100.46 ± 3.05 b	0.00	0.00	0.02
	B	26.24 ± 0.87 d	28.25 ± 0.21 d	37.52 ± 4.26 d	34.52 ± 1.32 d			

Results are shown as mean ± standard deviation (SD); means followed by the same letter (a,b,c,d) in a row A and B for the same low molecular weight carbohydrate are not significantly different (*p* < 0.05); d.m. dry matter; A—values before in vitro digestion (mg/g d.m.); B—values after in vitro digestion (mg/g d.m.).

## Data Availability

The data in this study are available on request from the author.

## Sample Availability

Not available.
